# It Is Not All about Alkaloids—Overlooked Secondary Constituents in Roots and Rhizomes of *Gelsemium sempervirens* (L.) J.St.-Hil

**DOI:** 10.3390/plants13162208

**Published:** 2024-08-09

**Authors:** Lilo K. Mailänder, Khadijeh Nosrati Gazafroudi, Peter Lorenz, Rolf Daniels, Florian C. Stintzing, Dietmar R. Kammerer

**Affiliations:** 1 Department of Analytical Development and Research, Section Phytochemical Research, WALA Heilmittel GmbH, Dorfstraße 1, DE-73087 Bad Boll/Eckwälden, Germany; khadijeh.nosrati-gazafroudi@wala.de (K.N.G.);; 2 Department of Pharmaceutical Technology, Tübingen University, Auf der Morgenstelle 8, DE-72076 Tübingen, Germany

**Keywords:** depsides, phenolic glycerides, mass spectrometry, natural products

## Abstract

*Gelsemium sempervirens* (L.) J.St.-Hil. is an evergreen shrub occurring naturally in North and Middle America. So far, more than 120 alkaloids have been identified in this plant in addition to steroids, coumarins and iridoids, and its use in traditional medicine has been traced back to these compound classes. However, a comprehensive phytochemical investigation of the plant with a special focus on further compound classes has not yet been performed. Therefore, the present study aimed at an extensive HPLC-MS^n^ characterization of secondary metabolites and, for the first time, reports the occurrence of various depsides and phenolic glycerides in *G. sempervirens* roots and rhizomes, consisting of benzoic and cinnamic acid derivatives as well as dicarboxylic acids. Furthermore, mono- and disaccharides were assigned by GC-MS. Applying the Folin–Ciocalteu assay, the phenolic content of extracts obtained with different solvents was estimated to range from 30 to 50% calculated as chlorogenic acid equivalents per g dry weight and was related to the DPPH radical scavenging activity of the respective extracts. Upon lactic acid fermentation of aqueous *G. sempervirens* extracts, degradation of phenolic esters was observed going along with the formation of low-molecular volatile metabolites.

## 1. Introduction

**Figure 1:**
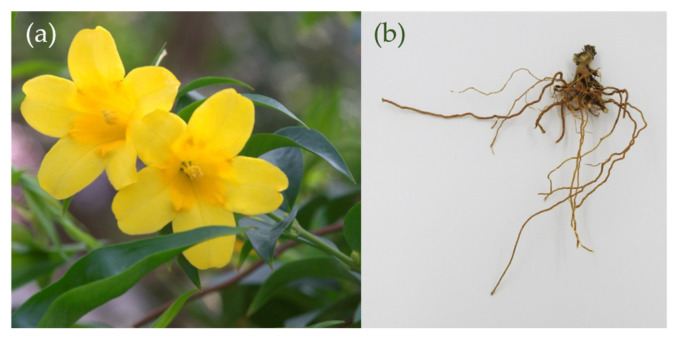
(**a**) Flowers of *G. sempervirens*. © Horst Arne Schneider. (**b**) Roots and rhizome of *G. sempervirens*. Photo: L. Mailänder.

*G. sempervirens* (L.) J.St.-Hil. (GS), also known as yellow jessamine, is an evergreen vine with a cylindrical rhizome and wiry roots [1]. With its fragrant yellow flowers, it is often used as an ornamental plant [1]. GS belongs to the genus *Gelsemium*, the only genus within the Gelsemiaceae plant family, which comprises only three highly toxic species [2]. While *G. elegans* (Gardner and Champ.) Benth. is distributed in Southern China and Southeast Asia, GS (Figure 1) and *G. rankinii* Small originate from North and Middle America [1,2]. Among these species, *G. elegans* has been phytochemically best studied, mainly by Asian research groups [3,4]. In contrast, fewer data are available on GS, while *G. rankinii* has been very scarcely investigated [2]. Previous publications have mainly focused on indole and oxindole alkaloids, of which more than 120 different constituents have been isolated from GS and *G. elegans* [2]. Based on their complex core structures, these have been classified into six different types: gelsemine, koumine, gelsedine, humantenine, sarpagine, and yohimbane. In addition, approximately ten steroids, twenty-five iridoids, and five coumarins have also been characterized in both species [5,6,7]. Further constituents such as phenolic acids, lignans, and saccharides have only been assigned in *G. elegans* using HPLC-MS [8,9].

Various pharmacological effects have been described for the three *Gelsemium* species. Different solvent extracts as well as isolated alkaloids have been found to exhibit anti-inflammatory, cytotoxic, and immunostimulatory activities and modulate noradrenaline and serotonin uptake, among many others [1,2]. The antinociceptive effects of the plant are often, but not exclusively, attributed to the alkaloids gelsemine and koumine and rely on the activation of spinal glycine receptors [10,11,12], which may also explain its neurotoxicity. Despite its toxicity, GS is traditionally applied as a medicinal plant for the treatment of neuralgia and fever [2] and was shown to have anxiolytic effects in mice [13].

Many of the phenolic compounds assigned in this study belong to the depsides, a substance class not previously described in the Gelsemiaceae. Depsides are defined as condensation products of two or more aromatic hydroxycarboxylic acids connected by an ester bond [14]. Depsides occur in many lichen species, where they are mainly composed of methyl- or alkyl-substituted dihydroxybenzoic acids but have also been found in fungi and higher plants [14]. Various interesting bioactivities, such as analgesic, antimalarial, neuroprotective, and wound healing activities, have been demonstrated for lichen depsides [15]. Rosmarinic acid, an ester of caffeic acid and 3,4-dihydroxyphenyllactic acid, is one of the best-studied non-lichen depsides, which was first isolated from rosemary (*Rosmarinus officinalis*) in 1958 [16]. Its antioxidant and anti-inflammatory activities have been exploited for the treatment of inflammatory diseases such as colitis or arthritis [17]. It is, however, sensitive to oxidation and easily degraded by fermentation or digestion processes [18].

To obtain information on the enzyme-catalyzed conversion of GS depsides and other constituents, aqueous GS extracts were subjected to a model fermentation using the ubiquitous lactic acid bacterium (LAB) *Lactiplantibacillus plantarum*. Contrary to most other LABs, *L. plantarum* is able to metabolize phenolic compounds such as ferulic, coumaric, caffeic, and gallic acids [19,20]. This metabolism normally comprises an esterase activity followed by decarboxylation reactions [21] resulting in the production of, e.g., ethyl or vinyl phenols or pyrogallol [19]. These volatile metabolites are also known from red wine [22], for example.

To the best of our knowledge, no comprehensive phytochemical analysis of GS roots and rhizomes with a focus on non-alkaloid metabolites has been reported so far. Consequently, this study aimed to expand the phytochemical knowledge of the Gelsemiaceae plant family and provide new perspectives on composition, antioxidant activity, and the fermentative metabolism of GS root and rhizome constituents. Therefore, an exhaustive extraction of the phytoconstituents with solvents of different polarity, i.e., dichloromethane, ethyl acetate, and *n*-butanol, was performed followed by thorough HPLC-DAD-MS^n^ and GC-MS analyses. In this way, both primary and secondary metabolites were assigned, with the focus of this study being clearly on the latter. The presented results may form a basis for further pharmacological studies supporting and potentially expanding the aforementioned medicinal applications, although the toxicity of the plant limits its use.

## 2. Results

### 2.1. Estimation of Total Phenolic Contents by Folin–Ciocalteu Assay

**Figure 2:**
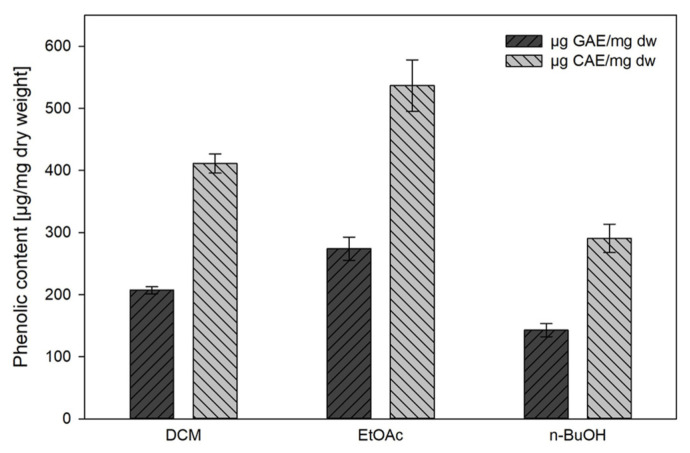
Total phenolic contents of dichloromethane (DCM), ethyl acetate (EtOAc), and *n*-butanol (*n*-BuOH) extracts of *G. sempervirens* roots and rhizomes. Results are expressed as µg gallic acid equivalents (GAE)/mg dry weight (dw) and µg chlorogenic acid equivalents (CAE)/mg dw, respectively; mean ± SD; *n* = 3.

The Folin–Ciocalteu colorimetric assay was applied to estimate the phenolic contents of the GS extracts. Absorbance values and calibration data are displayed in the Supporting Information (Part I). Gallic and chlorogenic acids were used for calibration purposes, and total phenolic contents were calculated as gallic acid equivalents (GAE) or chlorogenic acid equivalents (CAE). As displayed in Figure 2, GAE and CAE differed by almost 50%. Ethyl acetate (EtOAc) extracts showed the highest phenolic contents of 536.6 µg CAE/mg dry weight and 274.0 µg GAE/mg dry weight, respectively. The lowest phenolic contents were determined in *n*-butanol (*n*-BuOH) extracts, while dichloromethane (DCM) extracts ranged in between.

### 2.2. DPPH Radical Scavenging Activity of GS Extracts

**Figure 3:**
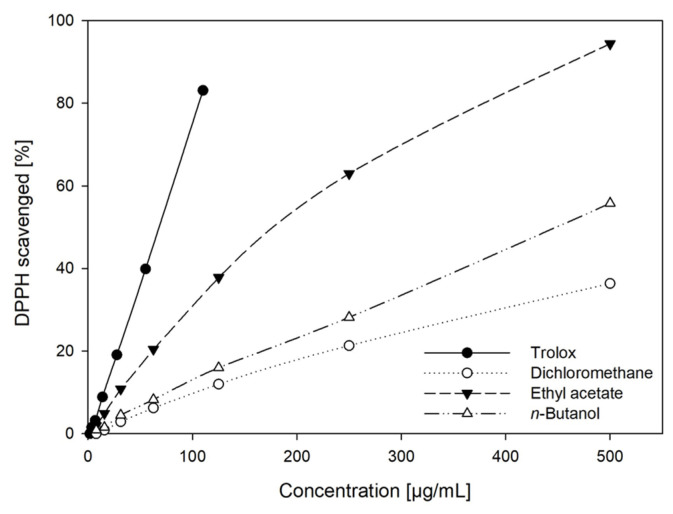
Percentage of DPPH scavenged by different solvent extracts from *G. sempervirens* roots and rhizomes in comparison to trolox as reference compound.

In addition to their phenolic contents, the 2,2-diphenyl-1-picrylhydrazyl (DPPH) scavenging activities of the aforementioned extracts and trolox as reference compounds were determined spectrophotometrically at 516 nm (Supporting Information Part II). The percentage of scavenged DPPH was calculated relative to the maximum amount scavenged and is displayed in Figure 3. The correlation between extract concentration and DPPH scavenging activity was linear for trolox (r^2^ = 1) and the BuOH extract (r^2^ = 0.99) in the entire concentration range examined. For the EtOAc and DCM extracts, linearity was only found at concentrations < 100 µg/mL. Generally, EtOAc extracts exhibited the highest radical scavenging activities. While the BuOH extract had the least total phenolic content, its antioxidant activity was stronger than that of the DCM extract. This is presumably due to differences in the phenolic composition of both extracts as our analyses have shown (Section 2.3 and Section 2.4).

### 2.3. Analysis of Low Molecular Constituents by GC-MS

**Figure 4:**
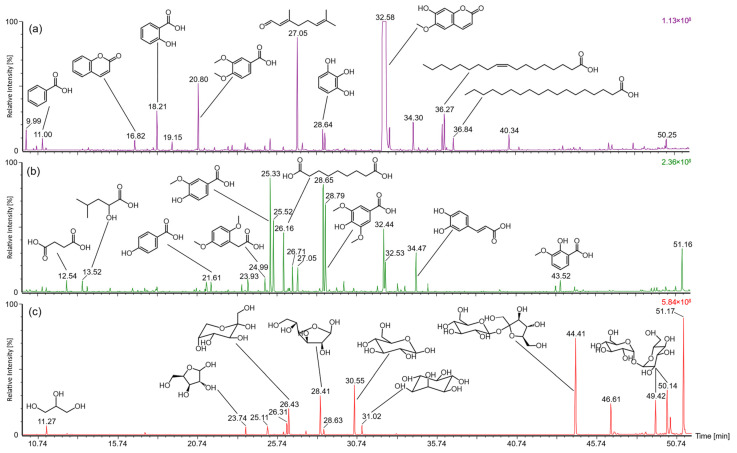
GC-MS profiles of secondary constituents in (**a**) dichloromethane, (**b**) ethyl acetate, and (**c**) *n*-butanol extracts after silylation. Compound assignment is presented in Table 1.

DCM, EtOAc, and BuOH extracts were analyzed by GC-MS after the silylation of individual constituents with BSTFA (Figure 4). For characterizing individual compounds, the obtained mass spectra (Table 1) were compared with the NIST database (National Institute of Standards and Technology, match factor > 800) and with MassBank Europe (massbank.eu, version 2.2.3). In DCM extracts, scopoletin (t_R_ 32.6 min) was by far the most abundant component, followed by citral (t_R_ 27.1 min). A variety of low molecular phenolic substances were eluted in a retention time range of 13–30 min. Among these, the three most abundant signals were attributed to coumarin (t_R_ 16.6 min), salicylic acid (t_R_ 18.2 min), and veratric acid (t_R_ 20.8 min). Between 34 and 37 min, adenine (t_R_ 34.3 min) and various fatty acids were eluted. Among the latter, linoleic, oleic, and stearic acids were the predominant compounds assigned based on their specific mass spectra. At higher retention times, mass spectrometric investigations indicated the presence of sterols. However, with match factors < 600, their exact identity could not be clarified. Various dicarboxylic and phenolic acids as well as scopoletin were characterized as main constituents in the chromatograms of EtOAc extracts. Finally, the polar BuOH extracts mainly contained a number of saccharides. Pentoses such as xylose and arabinose and hexoses such as fructose and glucose were eluted between 23 and 31 min. Disaccharides such as sucrose or trehalose were detected between 44 and 52 min. Furthermore, for aglycone analysis, the components of EtOAc and BuOH extracts were hydrolyzed with hydrochloric acid. The corresponding results can be found in the Supporting Information (Part III).

### 2.4. Analysis of Polar Secondary Constituents by HPLC-DAD-MS^n^

**Figure 5:**
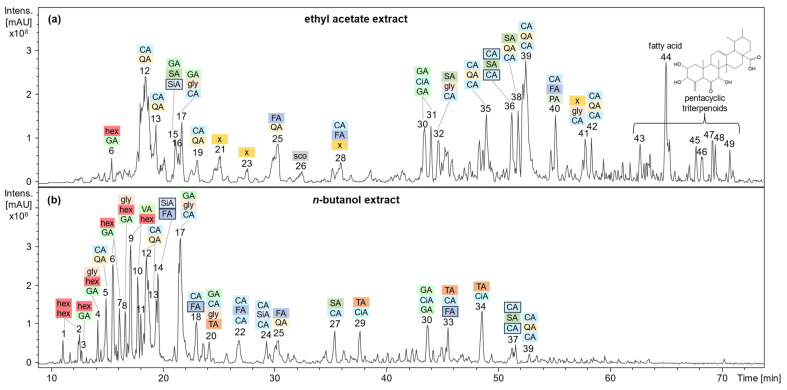
HPLC-MS^n^ base peak chromatograms of an (**a**) ethyl acetate and (**b**) *n*-butanol extract of dried *G. sempervirens* roots and rhizomes showing the occurrence of di- and tridepsides. The most abundant monomeric constituents are designated as follows: CA caffeic acid; CiA cinnamic acid; FA ferulic acid; GA gallic acid; gly glycerol; hex hexose; PA protocatechuic acid; QA quinic acid; SA syringic acid; sco scopoletin; SiA sinapinic acid; TA tartaric acid; VA veratric acid; x minor constituent (see Table 2); dihydro derivatives are displayed in bordered boxes.

Applying HPLC-DAD-ESI-MS^n^ in negative ionization mode, a variety of mostly phenolic substances were characterized based on the mass-to-charge ratios (*m/z*) of their precursor and fragment ions in comparison to the constituents and aglyca assigned in EtOAc and BuOH extracts by GC-MS. Moreover, for tentative structure elucidation, the fragmentation behavior of individual components in combination with UV spectral characteristics was compared to data published in the literature. A plethora of substances was assigned to depsidic structures, i.e., esters of two or three phenolcarboxylic acids, as displayed in Figure 5. Comprehensive HPLC-DAD-MS^n^ characteristics are displayed in Table 2.

Hydroxybenzoic acids such as gallic acid and its derivatives are characterized by two marked UV maxima at around 215 and 270 nm. Hydroxycinnamic acids such as ferulic and caffeic acids typically exhibit absorption maxima at approx. 220 and 320 nm [23]. The coumarin scopoletin has a characteristic UV spectrum with maxima at 207, 228, 296, and 342 nm [24]. However, at low analyte concentrations, UV spectra become less conclusive due to poor signal intensities.

**Figure 6:**
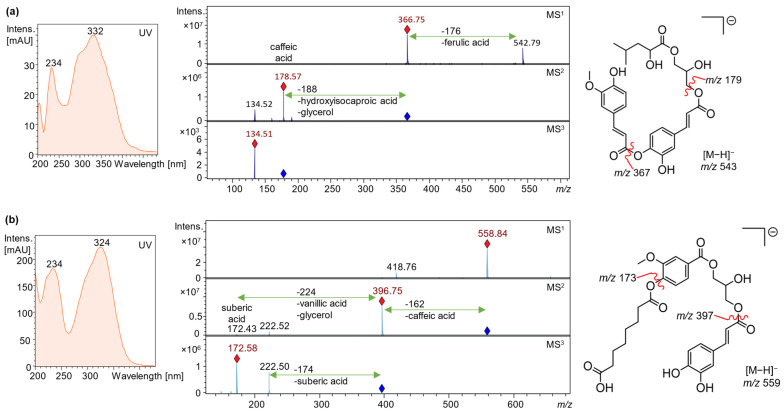
UV spectral and mass spectrometric structure assignment exemplified for compounds **28** (**a**) and **41** (**b**).

The characterization of phenolic esters and depsides was based on their molecular masses and neutral losses upon fragmentation and is exemplified in Figure 6 for compounds **28** and **41**. The most frequent molecular ions [M−H]^−^ were detected at *m/z* 149 (tartaric acid), 169 (gallic acid), 179 (caffeic acid or hexose, distinguishable by the UV spectrum), 191 (quinic acid), 193 and 195 (ferulic and dihydroferulic acid) and 197 (syringic acid), the corresponding neutral losses were 17 Da less [25,26,27,28,29,30]. Interestingly, various neutral losses (compounds **4**, **8**, **16**, **17**, **20**, **28**, **32**, **41**) pointed to constituents being composed of a phenolic acid moiety and glycerol [26,31], which was also detected by GC-MS. In accordance with the literature, we assumed that the phenolic acids are esterified with the primary hydroxy groups of glycerol as a consequence of their higher reactivity [31,32].

In the following section, compound assignment is exemplified for some representative substances. Compound **4** (t_R_ 14.2 min) exhibited a neutral loss of 242 Da in the MS^2^ experiment, which may be due to glycerol (92−17−17) and methyl gallic acid (184) moiety, producing a hexoside fragment ion (*m/z* 179). An isomer of this compound was eluted after 16.6 min (**8**). The compounds eluting next, **9** and **10** (t_R_ 17.1 and 17.7 min), revealed similar fragmentation patterns. A loss of 64 Da in the first fragmentation step pointed to sulfite or furan derivatives, the subsequent loss of 164 Da may be due to veratric, i.e., dimethoxybenzoic, acid, which has also been detected by GC-MS. As above, the MS^3^ base peak at *m*/*z* 179 indicated hexosides. Then, compound **16** (t_R_ 21.4 min), exhibiting an *m/z* at 505, showed a base peak at *m/z* 145 in the MS^2^ experiment which corresponds to [M−H−360]^−^, possibly [M−H−gallic acid−dihydrosinapinic acid]^−^. This is equivalent to the molar weight of ethylsuccinic or methylglutaric acid, both being aliphatic dicarboxylic acids. As a neutral loss of succinic acid (100 Da) may be assumed from the MS^1^ spectrum and succinic acid was also found by GC-MS, the substance was characterized as ethylsuccinyl-galloyl-dihydrosinapic acid. A neutral loss of 226 Da (gallic acid + glycerol) indicated compound **17** (t_R_ 21.7 min) to be a gallic and caffeic acid glyceride and compound **20** (t_R_ 24.1 min) to be a tartaric acid ester thereof. In contrast, compound **27** (t_R_ 35.4 min) had an [M−H]^−^ ion at *m/z* 359 and showed a loss of caffeic acid in the first fragmentation step. This may point to either caffeoyl-dihydroxyphenyllactic, i.e., rosmarinic, or caffeoyl-syringic acid. The latter assignment appears more plausible due to accordance with the GC-MS analyses. Furthermore, compounds **29** and **34** (t_R_ 37.6 and 48.6 min) were detected as formic acid adducts [M−H+46]^−^ of the respective molecular ions. Based on the results of GC-MS analyses and the findings for compound **16**, the neutral loss of 128 Da (compound **29**) was assigned to ethylsuccinic acid, and 142 Da (compound **34**) to propylsuccinic acid. The neutral loss of 144 Da in the MS^3^ experiments may be due to a hydroxycoumarin or a methylcinnamic acid moiety (compounds **29**–**31**, **34**). However, due to the lack of distinct UV maxima >300 nm, which would be expected for coumarins, the latter was assumed. Compounds **30** and **31** (t_R_ 43.5 and 44.0 min) could not be unambiguously characterized. Losses of 168 and 182 Da, possibly methyl- and dimethylgallic (syringic) acid, yielded MS^2^ base peaks at *m/z* 327. This ion was further fragmented yielding an MS^3^ base peak at *m/z* 183 (neutral loss of 144 Da, see above), which may either be ascribed to trihydroxyphenylacetic or methylgallic acid. Finally, the molecular ions of compounds **43** (t_R_ 62.7 min) and **45**–**49** (t_R_ 67.7–70.9 min) indicated the presence of pentacyclic triterpenoids such as gelse-norursanes, which have been described earlier [9,33].

Alkaloids form the most intensely investigated group of secondary metabolites in *Gelsemium* species. Expectedly, these were only detected by LC-MS in positive ionization mode [4,8,9]. However, since these have been well investigated and described in the literature, they were outside the focus of the present study. For chromatograms and a peak list please refer to the Supporting Information (Part IV). As deduced from the intensities of the base peak chromatograms, the alkaloid concentrations were higher in BuOH than in EtOAc extracts (Supporting Information Figure S4.1). In contrast, the latter were characterized by a more complex profile of individual compounds, particularly in a later retention time range. Gelsemine, N-methylgelsedilam, gelsemicine, and sempervirine were the main alkaloids detected in the BuOH extracts.

### 2.5. Metabolism of Phenolic Constituents upon Lactic Acid Fermentation

During the experiments, the progressive formation of a pleasant and spicy flavor of aqueous GS extracts was noticed. This observation is presumably due to the metabolic conversion of phenolic compounds caused by the microbial flora or endogenous plant enzymes after cell decompartmentation. Interestingly, the volatile compound formation was accelerated by inoculating the suspended plant material with *L. plantarum*, going along with a pH drop from 4.9 to 3.4 within three days as a result of lactic acid formation.

**Figure 7:**
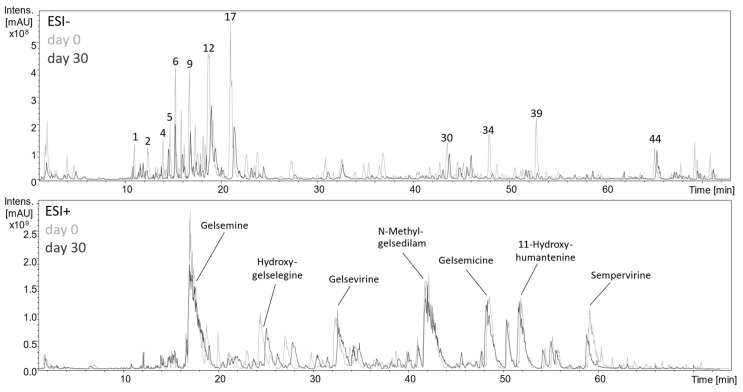
HPLC-MS^n^ base peak chromatograms of fermentation samples on day 0 (light grey) and 30 (dark grey) were recorded in negative (ESI−; top) and positive (ESI+; bottom) ionization mode. Peak numbers correspond to Table 2, LC-MS data of the alkaloids can be found in the Supporting Information (Table S4.1).

It is well known, that depsides and other esters are unstable upon enzymatic digestion and are rapidly hydrolyzed [18,34]. Accordingly, the HPLC-MS^n^ base peak chromatogram (Figure 7) showed a marked degradation of phenolic compounds, especially depsides and glycerides, within 30 days of lactofermentation. As can be deduced from the UV traces (Supporting Information Figure S4.2), the chlorogenic acid content decreased by almost 50% within seven days but then slowed down with declining microbial viability. While the caffeic acid content increased correspondingly, the coumarin scopoletin was obviously not metabolized. Except for a slight decrease in gelsemine and sempervirine contents, only minor changes were monitored in the alkaloid spectrum.

**Figure 8:**
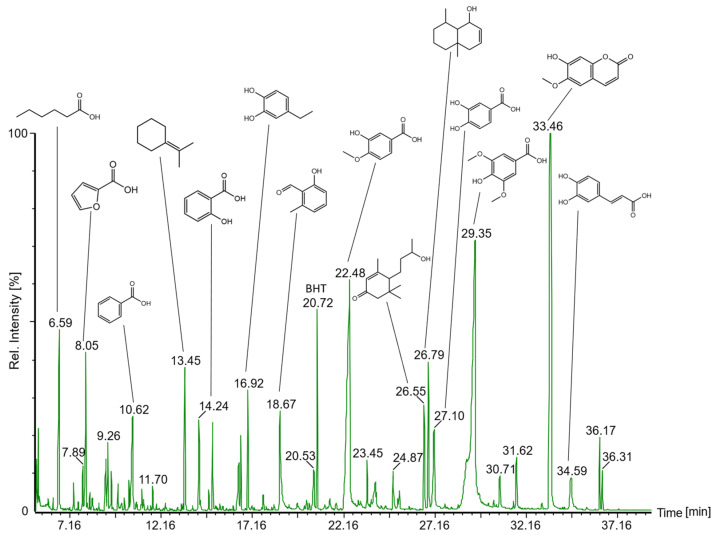
GC-MS total ion current chromatogram of volatile compounds extracted with diethyl ether from six months old aqueous fermented *G. sempervirens* extracts.

For analyzing the volatile constituents contributing to the intense smell of fermented aqueous GS extracts, these were extracted with diethyl ether and injected into the GC-MS system without prior derivatization. As described for DCM and EtOAc extracts, benzoic, salicylic, isovanillic, azelaic, protocatechuic, syringic, ferulic, and caffeic acids were assigned (Figure 8, for mass spectral data, see Supporting Information Table S3.2). In addition, aliphatic compounds such as propylcyclohexene, methylethylidene-cyclohexane, and dimethyloctene were assigned based on a comparison of mass spectral data with the NIST database. Among oxygen-containing metabolites, ethylcatechol, hydroxy-methylbenzaldehyde, and the sesquiterpenoid oxo-*α*-ionol were detected. Two isomeric compounds at retention times of 36.2 and 36.3 min, exhibiting a molecular mass of 222 Da, could not be further characterized. However, the molecular weight could indicate a hydroxy-dimethoxy-coumarin [35].

## 3. Discussion

### 3.1. Phenolic Content and Antioxidant Activity

The Folin–Ciocalteu (FC) and the DPPH assay are both used for assessing the antioxidant activity of plant extracts [36]. The FC assay corroborated the high content of phenolic constituents in GS extracts. More precisely, when calculated as chlorogenic acid equivalents, the dry matter of EtOAc extracts was composed of about 50% phenolic substances, the corresponding values of DCM and BuOH extracts amounted to 40% and 30%, respectively. However, it should be kept in mind that due to the unspecific redox reaction of the Folin assay, it may only be regarded as a semiquantitative method and rather as an indication of the reductive potential of the sample [37,38]. Although phenolic substances generally have strong antioxidant effects, the extent markedly depends on the molecular structure, e.g., the number of phenolic hydroxyl groups [37]. Consequently, the results calculated as gallic and chlorogenic acid equivalents differed by almost 50%. This phenomenon is well known and is due to the higher reducing capacity of a galloyl group compared to catechol or hydroxycinnamic acid groups and has been documented in the literature [37,39]. These effects were also reflected by the fact that despite higher phenolic contents of DCM extracts as deduced from the FC assay, their DPPH radical scavenging activity was weaker than that of BuOH extracts. Accordingly, differences in the phenolic profiles of the extracts could be shown in our analyses. Furthermore, while trolox and BuOH extracts showed a linear correlation between the percentage of scavenged DPPH and concentration in the entire concentration range tested, the curves of EtOAc and DCM flattened at concentrations > 100 µg/mL. This indicates that not only concentration but also the exact chemical composition affects the characteristics of such extracts and that concentration-dependent interactions between individual components may occur.

### 3.2. Phytochemical and Bioactivity Profiling of G. sempervirens Roots and Rhizomes

Saccharides were detected in the GC-MS chromatograms of crude BuOH extracts as well as in the aqueous residues after solvent partitioning. Rhizomes often contain high amounts of starch since they commonly serve as storage organs [40]. However, the extraction procedure and derivatization prior to analysis discriminate oligomeric and polymeric saccharides. This is the reason why only low molecular saccharides were covered in the present analysis. Among these, various ubiquitous pentoses and hexoses as well as disaccharides could be assigned. The occurrence of significant amounts of sugars in rhizomes is also known from other plants such as *Polygonatum* species [29]. Interestingly, while only saccharides were characterized in crude BuOH extracts, various phenolic constituents were detected after hydrolysis with hydrochloric acid and extraction with ethyl acetate. This indicates that part of the phenolics naturally occur in bound forms but can be released by enzymatic or acidic cleavage. In contrast, chromatograms of EtOAc extracts were comparable prior to and after hydrolysis in terms of peak profile and compound assignment.

Among phenolics, a variety of benzoic and cinnamic acid derivatives were assigned via GC-MS analysis, with vanillic, gentisic, syringic, and caffeic acids being the main phenolic acids in both, EtOAc and BuOH extracts. Furthermore, coumarins, fatty acids, dicarboxylic acids, saccharides, and glycerol were assigned based on comparison with the NIST database. A number of these substances such as scopoletin, vanillic acid, and chlorogenic acid have previously been assigned in *G. elegans* [8,9,41], which in combination with the alkaloids emphasizes the phytochemical similarity of the two *Gelsemium* species despite their origin from different continents [1]. The monoterpene citral, a mixture of the isomers geranial and neral, presumably contributes to the smell of GS roots but has not been detected in its flowers so far [42].

The LC-MS investigations performed in the present study expanded the knowledge of the complex composition of the phenolic profile, as not only monomers but also higher molecular weight compounds can be detected using this technique. The assignment of most substances was based on their mass spectrometric behavior and UV spectra, which was aligned with the findings for the monomers obtained by GC-MS in crude and hydrolyzed EtOAc and BuOH extracts. Many of the compounds characterized according to this procedure belong to the depsides, i.e., esters of two or more phenolic acids [43]. Depsides have particularly been found in many lichen [15,44] and fungal [14] species, where they mostly consist of orsellinic, i.e., 2,4-dihydroxy-6-methylbenzoic, acid [14]. However, depsides of phenolic acids without methyl substitution of the aromatic ring have also been found in various higher plants such as aronia [45], rosemary [18], sage [46], or pineapple [47], thus across a wide range of plant families. The manifold bioactivities described for this compound class include cytotoxic, antimicrobial, analgesic, hepato-, nephro-, and neuroprotective as well as anti-inflammatory effects [15,17,46], which renders GS extracts potentially interesting from a pharmacological viewpoint.

Phenolic glycerides are another substance class characterized for the first time in the Gelsemiaceae in the present study. They have, however, also been detected in other plant families such as the Liliaceae [48], Bromeliaceae [49], and Asparagaceae [26], and are characteristic of propolis [31,50]. Like other phenolic substances, they exhibit marked antioxidant and anti-inflammatory activities [51,52]. Thus, while the toxicity of GS is attributed to alkaloids, the described inflammation, and eczema-reducing activity [2] may also be due to the phenolic constituents herein or due to an interplay of the different compound classes in the complex mixture.

Nor-ursane type triterpenoids such as the gelse-norursanes assigned in this study have been isolated from representatives of approx. 15 different plant families, mostly but not exclusively occurring in tropical and subtropical regions [33]. The natural habitat of GS from Florida to Virginia also fits in this climate zone [2]. Interesting bioactivities have been described for similar pentacyclic triterpenoids, for example, antidiabetic effects due to inhibition of the insulin-resistance-promoting enzyme tyrosine proteinase [53]. In addition, hepatoprotective effects [54] as well as cytotoxicity against leukemia, liver, breast, and colon cancer cells [55,56] were demonstrated.

### 3.3. Fermentation of Aqueous GS Root Extracts

The rapid formation of an aromatic odor within one week of fermentation in combination with a marked pH decrease indicated that the growth and viability of *L. plantarum* were not affected by the high alkaloid contents of GS. GC-MS analyses of the volatile compounds in fermented GS extracts revealed the presence of various low-molecular phenolic acids and metabolites thereof. As an example, 4-ethylcatechol may be produced from hydroxycinnamic acids by *L. plantarum* and is a well-known off-flavor component in various fermented foods [57]. *γ*-Amylbutyrolactone (*γ*-nonalactone) is an odor-active compound also found in whiskey [58], the sesquiterpenoid oxo-*α*-ionol is a metabolite produced by yeasts during winemaking [59]. However, it was not investigated if only a few character impact compounds account for the spicy smell of the fermented extracts, or if it is caused by a more complex variety of compounds detected in larger concentrations.

Using HPLC-MS^n^ analysis in negative ionization mode, a degradation of saccharides, glycerides as well as depsides was observed as can be deduced from the base peak chromatograms. This is not surprising, since several esterases have been described in *L. plantarum* [21,60], which, among others, leads to the release of further compounds that may serve as substrates for bacterial metabolization. In contrast, only minor changes were detected in the alkaloid spectrum analyzed in positive ionization mode. The pH decline from 4.9 to 3.4 upon fermentation may have a stabilizing effect on the alkaloids, as they are protonated at lower pH values, increasing their solubility in water. Still, the decrease in gelsemine and sempervirine is most likely due to their poor solubility in aqueous systems, which leads to precipitation. Accordingly, both components were also found in the sediment formed during storage, after extraction of the latter with methanol. To conclude, fermentation appears to be a suitable method for the extraction of secondary metabolites from the plant matrix of GS as well as for preserving the obtained aqueous extracts, despite the metabolic degradation, particularly of phenolic constituents. The obtained extracts may, among others, be used for pharmaceutical applications, although their toxicity due to the alkaloid fraction must be considered.

## 4. Materials and Methods

### 4.1. Chemicals and Reagents

Acetone, acetonitrile, *n*-butanol (BuOH), chloroform, dichloromethane (DCM), ethyl acetate (EtOAc), hydrochloric acid, methanol (MeOH), methyl-*tert*-butyl ether (MTBE), sodium carbonate and sodium sulfate were purchased from Chemsolute (Th. Geyer GmbH & Co. KG, Renningen, Germany). Diethyl ether, gallic acid monohydrate (GA), and lactose were obtained from Carl Roth GmbH & Co. KG (Karlsruhe, Germany). *N,O*-Bis(trimethylsilyl)-trifluoroacetamide (BSTFA), dimethylformamide (DMF), and 2,2-diphenyl-1-picrylhydrazyl (DPPH) were from Sigma-Aldrich (St. Louis, Missouri, USA), formic acid from Fluka (Sigma Aldrich, St. Louis, MO, USA). Trolox was purchased from Cayman Chemical Company (Ann Arbor, MI, USA), and chlorogenic acid hemihydrate (CA) from Alfa Aesar (Karlsruhe, Germany). Folin–Ciocalteu’s reagent was from Merck KGaA (Darmstadt, Germany). Ultrapure water was produced with an ELGA Purelab Classic system (High Wycombe, UK) and used for all experiments.

### 4.2. Plant Material and Extraction

**Figure 9:**
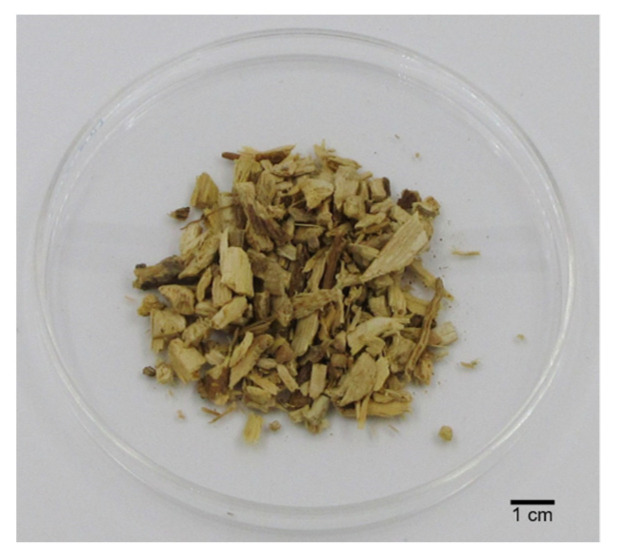
Dried *G. sempervirens* plant material used in this study.

Dried and cut *G. sempervirens* roots and rhizomes (Figure 9) were obtained from a commercial supplier (Albert Stephan export–import, Zweibrücken, Germany). A sample was deposited at the herbarium of the Institute of Botany, University of Hohenheim, Stuttgart (voucher number: HOH-022975).

For the extraction, 35 g plant material was mixed with 500 mL 70% acetone (*v*/*v*), bubbled with nitrogen for 5 min, and minced by Ultra-Turrax treatment (IKA Werke GmbH and Co. KG, Staufen, Germany; 3 min, 17,000 rpm). After another 10 min bubbling with nitrogen, the slurry was stored at 4 °C overnight. The mixture was then filtered over Celite^®^ (Carl Roth GmbH & Co. KG, Karlsruhe, Germany) and the filter cake was re-extracted analogously. Then, the two filtrates were combined, the acetone was removed by rotary evaporation, and the residual aqueous phase was extracted successively with 3 × 100 mL of DCM, EtOAc, and *n*-BuOH. DCM and EtOAc extracts were dried over anhydrous sodium sulfate and filtered through filter paper (Whatman™ qualitative filter paper 2, GE Healthcare, Buckinghamshire, UK). Solvents were removed in vacuo and the dry residues were used for further experiments. Extraction was performed in duplicate.

For aglycone analyses, 50 mg of EtOAc or BuOH extracts were dissolved in 1 N hydrochloric acid (20 mL) and kept at 105 °C for 1 h. Aglycones were then extracted with 2 × 30 mL EtOAc, the organic phase was dried over sodium sulfate, filtered, and the solvent removed by rotary evaporation.

### 4.3. Fermentation Experiments

For the fermentation experiments, 20 g of dried roots were mixed with 500 mL of water containing 0.75% (*w*/*v*) lactose as substrate for microbial fermentation. The material was minced using an UltraTurrax (2 min, 17,000 rpm). This suspension was then inoculated with 1 mL of *Lactiplantibacillus plantarum* (previously *Lactobacillus plantarum*, GenBank accession number: MK841313.1; sequence length: 1083 base pairs; closest relative in National Center for Biotechnology Information: *Lactobacillus plantarum* strain 2.7.17, MK611349.1; similarity 100%) in MRS broth (5 × 10^8^ CFU/mL). After three days at 33 °C, the slurry was filtered through a cotton cloth yielding a turbid solution and the filtrate was kept in glass bottles at room temperature in the dark. After one week the turbid solution was filtered through filter paper yielding a clear solution, which was stored for a minimum of six months. Three temporally independent fermentations were conducted, each in duplicate or triplicate.

### 4.4. Estimation of the Total Phenolic Content by Folin–Ciocalteu Assay

The dried DCM, EtOAc, and BuOH extracts were dissolved in MeOH (250 µg/mL). Gallic acid (GA) and chlorogenic acid (CA) were used as reference substances in concentrations ranging from 3.5 to 55 µg/mL (GA) and 10–160 µg/mL (CA), respectively. A 20 µL amount of sample or reference solution and 40 µL of Folin–Ciocalteu’s reagent were mixed on a 96-well plate. The plate was shaken in the reader for one minute, and subsequently, 160 µL of sodium carbonate solution (700 mM) was added. After incubation (37 °C, 30 min), the absorbance at 765 nm was measured using a multiplate reader (Epoch2, Agilent Technologies Inc., Santa Clara, CA, USA). Calibration equations of GA and CA were calculated by plotting the absorbance values against the concentrations. The total phenolic content of the samples was then calculated as GA or CA equivalents [mg/g dry weight] by inserting the sample absorbance values into the regression equations. Analyses were performed in triplicate. Absorbance values and calibration data are provided in the Supporting Information (Part III).

### 4.5. Determination of the DPPH Radical Scavenging Activity

The DCM, EtOAc, and BuOH extracts were dissolved in MeOH and diluted to seven concentrations between 7.8 and 500 µg/mL. Trolox (1.7 to 110 µg/mL) was used as reference compound. 180 µL of DPPH solution (100 µM in MeOH) was then added to 20 µL of the test or reference solution or methanol as a blank sample in a 96-well plate. The plate was incubated at 37 °C for 45 min and then analyzed colorimetrically at 516 nm using a multiplate reader Epoch2. The percentage of scavenged DPPH was calculated from the maximum and sample absorbances (A) using the formula

DPPH scavenged [%] = (A_max_ − A_sample_)/A_max_ × 100%.

Analyses were performed in triplicate. Absorbance values are provided in the Supporting Information (Part IV).

### 4.6. GC-MS Analyses

For GC-MS analyses, crude DCM, EtOAc, and BuOH extracts and their aglycones (5–10 mg) were dissolved in DMF (500 µL) and mixed with BSTFA (200 µL). The solution was heated to 105 °C for 15 min in order to obtain trimethylsilyl derivatives of individual compounds.

For the extraction of volatile aroma composites from the fermented solutions, 40 mL of each sample was extracted with 2 × 10 mL diethyl ether. The solvent was removed under reduced pressure, and the residue was dissolved in 1 mL methyl-*tert*-butyl ether (MTBE) and directly used for analysis.

GC-MS analyses were conducted with a PerkinElmer Clarus 500 gas chromatograph (PerkinElmer, Inc., Shelton, CT, USA) coupled to a single quadrupole mass spectrometer operating in electron ionization (EI) mode at 70 eV according to [61]. Split injection (split ratio 30:1, injection volume 1.0 µL) was applied and a Zebron ZB-5MS capillary column (60 m × 0.25 mm i.d., 0.25 µm film thickness, 5% phenylpolysiloxane and 95% dimethylpolysiloxane coating; Phenomenex, Torrance, CA, USA) was used as a stationary phase. Helium served as carrier gas at a flow rate of 1 mL/min. The injector temperature was 250 °C, and the temperature of the column oven was 100–320 °C with a linear gradient of 4 °C/min. Data were acquired and processed using the software TurboMass (v.5.4.2, PerkinElmer, Inc., Waltham, MA, USA).

### 4.7. HPLC-DAD-ESI-MS^n^ Analyses

For HPLC analyses, EtOAc and BuOH extracts were dissolved in MeOH or water (5 mg/mL), respectively, and aqueous samples were directly injected after filtration through a syringe filter (perfect-flow RC, 0.45 µm, WICOM, Heppenheim, Germany).

Reversed phase high performance liquid chromatography was carried out as described previously [61]. In brief, an Agilent 1200 HPLC system (Agilent Technologies, Inc., Palo Alto, CA, USA) equipped with binary pump, micro vacuum degasser, autosampler, thermostatic column compartment and UV/VIS diode array detector (DAD); a Kinetex^®^ C18 reversed-phase column (2.6 µm particle size, 150 mm × 2.1 mm i.d., Phenomenex Ltd., Aschaffenburg, Germany); and a pre-column of the same material were used for chromatographic separation at 25 °C and a flow rate of 0.21 mL/min. 0.1% formic acid in water and acetonitrile were used as mobile phase.

For mass spectrometric detection, an HCTultra ion trap mass spectrometer (Bruker Daltonik GmbH, Bremen, Germany) with an ESI source was used. All extracts were analyzed in positive and negative ionization mode using a capillary voltage of + or −4000 V, respectively. The dry gas (N_2_) flow was 9.00 L/min, capillary temperature 365 °C, and nebulizer pressure 35 psi. MS^n^ data were generated by performing collision-induced dissociation (CID) experiments. The instruments were controlled by Agilent LC 3D systems (Rev. B01.03SR1 (204)) and Bruker Daltonics EsquireControl software (V7.1).

## 5. Conclusions

In the investigation presented here, secondary metabolites in GS roots and rhizomes were comprehensively characterized with a special focus on phenolic constituents. Applying the Folin–Ciocalteu assay, total phenolic contents of 411, 537, and 291 µg chlorogenic acid equivalents per mg dry weight were determined in DCM, EtOAc, and BuOH extracts, respectively. Accordingly, pronounced antioxidant activity was determined using the DPPH antioxidant assay. Interestingly, the correlation between concentration and DPPH scavenging activity was not strictly linear for EtOAc and DCM extracts, indicating concentration-dependent interactions between individual components.

The identity of the phenolic compounds was studied by GC-MS and HPLC-DAD-MS^n^ analyses and could mainly be assigned to depsides and phenolic glycerides consisting of various hydroxybenzoic, hydroxycinnamic, and dicarboxylic acids. A plethora of bioactivities have been reported for the aforementioned constituents, such as anti-inflammatory, analgesic, and neuroprotective action. They may therefore also contribute to the pharmacological effects described for GS, which have previously been attributed mainly to alkaloids.

Upon lactic acid fermentation with *L. plantarum*, depsides, glycerides, and other esters were rapidly degraded. Subsequently, the formation of low-molecular phenolic metabolites could be shown in GC-MS analyses. The obtained extracts remained microbially stable during the six-month period of investigation. The presented results expand the knowledge on the traditional medicinal plant GS and may open new perspectives of use, despite its toxicity limiting pharmaceutical applications.

## Figures and Tables

**Table 1:** Compound assignment of substances detected using GC-MS in (a) dichloromethane, (b) ethyl acetate, and (c) *n*-butanol extracts after silylation. Corresponding chromatograms are illustrated in Figure 4.

t_R_ [min]	Constituent (TMS Derivative)	MW [Da]	Fragment *m/z* (Intensity %)
(a) Dichloromethane			
10.0	2-Octenoic acid	214.1	214 (38), 199 (82), 124 (100), 109 (62), 73 (88), 55 (28)
11.0	Benzoic acid	194.3	194 (7), 179 (100), 135 (66), 105 (65), 77 (49)
16.8	Coumarin	146.1	146 (96), 118 (100), 89 (45), 75 (12), 77 (49)
18.2	Salicylic acid	282.5	267 (100), 209 (10), 73 (82)
19.2	Vanillin	224.3	224 (26), 209 (46), 194 (100), 73 (21)
20.8	Veratric acid	254.4	254 (44), 239 (100), 136 (93), 73 (90)
27.1	Citral	152.2	152 (53), 107 (26), 84 (73), 69 (100)
28.6	Pyrogallol	342.7	329 (36), 239 (34), 209 (21), 147 (49), 93 (32), 73 (100)
28.8	Syringic acid	342.5	342 (67), 327 (100), 312 (79), 297 (63), 253 (39), 73 (71)
32.6	Scopoletin	264.3	264 (48), 234 (100), 206 (37), 191 (8), 176 (10), 73 (36)
34.3	Adenine	279.5	294 (37), 279 (25), 264 (100), 73 (30)
36.2	Linoleic acid	352.3	337 (31), 129 (37), 117 (32), 95 (44), 81 (58), 73 (100), 55 (54)
36.3	Oleic acid	354.3	354 (2), 339 (51), 129 (68), 117 (91), 73 (100), 55 (63)
36.8	Stearic acid	356.3	356 (6), 341 (78), 132 (63), 117 (100), 73 (80), 55
40.3	Undefined sterol		440 (4), 369 (8), 225 (75), 130 (23), 93 (17), 73 (100)
50.2	Undefined sterol		386 (7), 371 (37), 281 (36), 269 (46), 207 (42), 73 (100)
(b) Ethyl acetate			
12.5	Succinic acid	262.4	247 (11), 147 (100), 73 (35)
13.5	2-Hydroxy-isocaproic acid	276.5	247 (66), 159 (82), 147 (47), 115 (19), 73 (100)
21.6	Salicylic acid	282.5	282 (22), 267 (38), 193 (18), 73 (100)
23.9	Suberic acid	318.6	303 (58), 213 (30), 147 (71), 73 (100), 69 (44), 55 (66)
25.0	2,5-Dimethoxy-phenylacetic acid	268.4	268 (16), 253 (32), 209 (38), 134 (25), 105 (36), 91 (29), 73 (100)
25.3	Vanillic acid	312.1	312 (57), 297 (100), 282 (34), 267 (67), 253 (51), 223 (51), 126 (31), 73 (49)
25.5	Gentisic acid	370.6	370 (4), 355 (100), 73 (60)
26.2	Azelaic acid	332.6	317 (51), 201 (52), 147 (26), 129 (31), 117 (29), 73 (100), 55 (48)
26.7	Protocatechuic acid	370.6	370 (49), 355 (26), 311 (21), 193 (100), 73 (53)
27.1	Citral	152.2	152 (42), 107 (23), 93 (23), 84 (67), 81 (29), 75 (60), 69 (100)
28.7	Pyrogallol	342.7	329 (27), 239 (29), 209 (18), 147 (46), 143 (34), 119 (28), 103 (28), 73 (100)
28.8	Syringic acid	342.5	342 (66), 327 (100), 312 (76), 297 (66), 253 (43), 149 (32), 141 (33), 73 (77)
32.4	Scopoletin	264.3	264 (50), 234 (100), 206 (40), 191 (8), 176 (11), 73 (31)
32.5	Vanillylmandelic acid	414.7	428 (64), 297 (100), 73 (83)
34.5	Caffeic acid	396.7	396 (71), 381 (19), 219 (100), 191 (16), 73 (79)
43.5	Methoxysalicylic acid	312.5	297 (100), 73 (48)
51.2	Saccharide derivative		331 (30), 253 (85), 217 (100), 204 (21), 147 (31), 103 (25), 93 (27), 73 (89)
(c) *n*-Butanol			
11.3	Glycerol	308.6	218 (19), 205 (58), 147 (90), 133 (20), 117 (33), 103 (31), 73 (100)
23.7	Xylose	438.8	217 (39), 204 (100), 191 (41), 147 (33), 73 (66)
25.1	Arabinose	438.8	217 (45), 204 (100), 191 (45), 147 (34), 73 (83)
26.3	Fructofuranose	541.1	437 (13), 217 (78), 147 (28), 73 (100)
26.4	Fructopyranose	541.1	437 (24), 217 (27), 204 (78), 147 (37), 73 (100)
28.4	Glucose	541.1	217 (18), 204 (100), 191 (50), 147 (24), 73 (62)
28.6	Galactose	541.1	329 (22), 239 (23), 217 (18), 204 (63), 191 (35), 147 (56), 143 (27), 73 (100),
30.6	Glucopyranose	541.1	217 (19), 204 (100), 191 (54), 147 (24), 73 (62)
31.0	Myo-Inositol	613.2	318 (23), 305 (32), 217 (70), 191 (27), 147 (48), 133 (33), 73 (100)
44.4	Sucrose	919.7	437 (18), 361 (100), 217 (43), 147 (25), 103 (19), 73 (81)
46.6	Unknown	394.5	394 (22), 351 (21), 323 (16), 134 (16), 108 (100), 73 (43)
49.4	Disaccharide	919.7	361 (94), 340 (38), 251 (38), 217 (33), 204 (20), 191 (28), 147 (31), 73 (100)
50.1	Disaccharide	919.7	373 (19), 217 (18), 204 (100), 147 (16), 73 (47)
51.2	Saccharide derivative		331 (29), 253 (85), 217 (100), 204 (22), 147 (26), 103 (23), 93 (24), 73 (83)

**Table 2:** HPLC-DAD-MS^n^ characteristics of individual secondary metabolites in ethyl acetate (EtOAc) and *n*-butanol (BuOH) extracts from *G. sempervirens* roots and rhizomes obtained in negative ionization mode. Corresponding chromatograms are illustrated in Figure 5.

Peak Number		t_R_ [min]	λ_max_ [nm]	Negative Ionization *m/z*			Compound Assignment
EtOAc ^a^	BuOH ^b^			MS^1^	MS^2^	MS^3^	
	1	11.1	-	421 ^c^, 411	225, 179 ^c^	161, 143 ^c^, 119	Saccharide
	2	12.4	204	393 ^d^	347 ^c^	185, 161 ^c^, 143	Saccharide
	3	12.5	ND ^e^	375 ^c^	213 ^c^	169 ^c^	Gallic acid hexoside
	4	14.2	206, 230, 308	421 ^c^	179 ^c^	143 ^c^	Methylgallic acid hexoside glycerol ester
	5	14.9	204, 324	353 ^c^	191 ^c^, 179	173 ^c^	4-*O*-Caffeoylquinic acid
6	6	15.5	238	375 ^c^	213, 169 ^c^	151, 125 ^c^, 109	Gallic acid hexoside
	7	16.1	252	375 ^c^	213 ^c^, 169	125, 107 ^c^	Gallic acid hexoside
	8	16.6	208, 254	421 ^c^	179 ^c^	143 ^c^, 119, 89	Methylgallic acid hexoside glycerol ester
	9	17.1	209, 254	407 ^c^, 397	343 ^c^	179 ^c^, 161, 143, 119	Veratric acid hexoside derivative
	10	17.7	204, 288	407 ^c^	343, 179 ^c^	143, 119, 89, 83	Veratric acid hexoside derivative
	11	18.0	204, 225, 290sh, 328	377 ^d^	331 ^c^	161 ^c^	Hydroxycoumarin and azelaic acid ester
12	12	18.5	218, 236, 312, 328	707, 353 ^c^	191	173, 127, 111, 93	5-*O*-Caffeoylquinic acid
13	13	19.4	220, 240sh, 290sh, 326	353 ^c^	191, 173 ^c^	127, 93	4-*O*-Caffeoylquinic acid
	14	19.6	224, 324	403 ^c^, 353	237, 195 ^c^	165, 151, 97	Dihydroferuloyl-dihydrosinapinic acid
15		21.1	216, 274	557 ^c^	197 ^c^	181, 153, 137 ^c^	Syringyl-galloyl-dihydrosinapinic acid
16		21.4	222, 296	505 ^c^, 405	145 ^c^		Succinyl-galloyl-dihydrosinapinic acid derivative
17	17	21.7	226, 302	405 ^c^	225, 179 ^c^	89 ^c^	Gallic and caffeic acid glyceride
	18	23.0	230, 326	403 ^d^	357, 195 ^c^, 179	151, 125 ^c^	Caffeoyl-dihydroferulic acid
19		23.1	230, 316	353 ^c^	191 ^c^	171, 127	3-*O*-Caffeoylquinic acid
	20	24.1	308	537 ^c^	311 ^c^	293, 233, 149 ^c^, 101	Caffeoyl-galloyl-tartaric acid glyceride
21		25.1	218, 316	391 ^f^, 195 ^c^	151 ^c^		Dihydroferulic or dimethylphenyl-acetic acid
	22	26.8	238, 324	517 ^c^	193 ^c^	176, 149, 134 ^c^	Dicaffeoyl-ferulic acid
23		27.6	226, 264	363 ^c^	315 ^c^, 272	300, 272, 256 ^c^	Methylellagic acid derivative
	24	29.3	238, 324	547 ^c^	367, 325, 295, 265, 223 ^c^	205, 163 ^c^	Dicaffeoyl-sinapinic acid
25	25	30.3	212, 324	367 ^c^	191 ^c^, 173	173 ^c^, 93	Feruloylquinic acid
26		32.4	207, 228, 296, 342	191 ^c^	176 ^c^		Scopoletin
	27	35.4	308	359 ^c^	197 ^c^	153 ^c^, 135, 109	Caffeoylsyringic acid
28		35.9	204, 234, 332	543, 367 ^c^	179 ^c^, 161	135 ^c^	Feruloyl-caffeoyl- hydroxyisocaproic acid glyceride
	29	37.6	224, 282	467 ^d^	421 ^c^, 293	293 ^c^, 191, 149	Tartaric and methylcinnamic acid ester
30	30	43.5	224, 282	495 ^c^	463, 327 ^c^	311, 183	Digalloylcinnamic acid derivative
31		44.0	222, 260sh, 286	509 ^c^	327 ^c^	183 ^c^	Digalloylcinnamic acid derivative
32		44.6	222, 264, 300	465 ^c^	433 ^c^	289, 271 ^c^, 179	Caffeoylsyringic acid glyceride
	33	45.5	228	549 ^d^	503 ^c^	371 ^c^, 161	Dihydroferuloyl-caffeoyltartaric acid
	34	48.6	232	481 ^d^	435 ^c^, 293	293 ^c^, 149	Tartaric and methylcinnamic acid ester
35		49.0	222, 235, 300sh, 326	515 ^c^	353 ^c^	191 ^c^, 179, 135	3,5-Di-*O*-caffeoylquinic acid
36		51.2	222, 282	539 ^c^, 515	523 ^c^, 341	197 ^c^	Di-dihydrocaffeoyl-syringic acid methyl ester
	37	51.5	224, 288	541 ^c^	523 ^c^	197 ^c^	Di-dihydrocaffeoyl-syringic acid methyl ester derivative
38		51.8	232, 292	533 ^c^	371 ^c^	197, 173 ^c^	Syringyl- caffeoyl-quinic acid
39	39	52.5	220, 240, 300sh, 326	515 ^c^	353 ^c^	173 ^c^	4,5-Di-*O*-caffeoylquinic acid
40		55.1	232, 324	491 ^c^	315 ^c^	153 ^c^	Feruloyl-caffeoyl-protocatechuic acid
41		57.8	234, 324	559 ^c^	397 ^c^	223, 173 ^c^	Caffeoyl-vanillyl-suberic acid glyceride
42		58.4	232, 290, 340	515 ^c^	353 ^c^	191 ^c^, 179, 173	3,4-Di-*O*-caffeoylquinic acid
43		62.7	232, 326	501 ^c^	483 ^c^, 465, 439	419 ^c^, 403	Gelse-norursane A
44		65.0	-	329 ^c^	211, 293, 229 ^c^, 171, 158		Trihydroxy-octadecenoic acid isomer
45		67.7	-	483 ^c^	419, 391 ^c^, 379, 203	321 ^c^	Gelse-norursane derivative
46		68.2	-	515 ^c^	471 ^c^	453 ^c^, 427	Gelse-norursane C derivative
47		69.1	-	485 ^c^	467 ^c^	437 ^c^, 355	Gelse-norursane B
48		69.4	-	485 ^c^	467, 405 ^c^	363 ^c^	Gelse-norursane B
49		70.9	-	469 ^c^	405 ^c^	375 ^c^	Gelse-norursane E

^a^ Signals in ethyl acetate extract, see Figure 2; ^b^ signals in *n*-butanol extract; ^c^ ion isolated for subsequent fragmentation; ^d^ formic acid adduct [M−H+HCOOH]^−^; ^e^ not detected; ^f^ dimeric ion [2M−H]^−^.

## Data Availability

Data is contained within the article and supplementary material.

## Supplementary Materials

The following supporting information can be downloaded at: https://www.mdpi.com/article/10.3390/plants13162208/s1, Table S1.1. Calibration data for the Folin–Ciocalteu assay obtained with gallic acid. Table S1.2. Calibration data for the Folin–Ciocalteu assay obtained with chlorogenic acid. Table S1.3. Total phenolic content of *G. sempervirens* DCM, EtOAc, and BuOH extracts (250 µg/mL) calculated as µg gallic or chlorogenic acid equivalents per mg dry weight. Table S2.1. Calibration data for the DPPH radical scavenging assay obtained with trolox. Table S2.2. DPPH radical scavenging capacity of the DCM extract at different concentrations. Table S2.3. DPPH radical scavenging capacity of the ethyl acetate extract at different concentrations. Table S2.4. DPPH radical scavenging capacity of the *n*-butanol extract at different concentrations. Figure S3.1. GC-MS total ion current chromatograms of hydrolyzed (a) *n*-butanol and (b) ethyl acetate extracts after silylation. Table S3.1. Aglycones detected in ethyl acetate and *n*-butanol extracts after hydrolysis and silylation using GC-MS. Compound assignment was achieved by comparison with the NIST database (match factor >800). Table S3.2. GC-MS data of volatile compounds extracted with diethyl ether from aqueous fermented samples. Compound assignment was achieved by comparison with the NIST database (match factor >800). Figure S4.1. HPLC-DAD-ESI-MS^n^ base peak chromatograms of (A) ethyl acetate and (B) n-butanol extracts of *G. sempervirens* roots and rhizomes recorded in positive ionization mode. Table S4.1. HPLC-DAD-MS^n^ characteristics of individual secondary metabolites in ethyl acetate and n-butanol extracts from *G. sempervirens* roots and rhizomes obtained in positive ionization mode. Figure S4.2. Stacked display of UV chromatograms (328–332 nm) of aqueous fermented *G. sempervirens* extracts within 30 days of fermentation. References [62,63] are cited in the supplementary materials.
