# Supplementary material for: It Is Not All about Alkaloids—Overlooked Secondary Constituents in Roots and Rhizomes of Gelsemium sempervirens (L.) J.St.-Hil
Source: Plants (Basel). 2024 Aug 9;13(16):2208. doi: 10.3390/plants13162208 (PMC11358907; doi:10.3390/plants13162208)
Supplement: Supplementary file 1 [file plants-13-02208-s001.zip › plants-3069496-supplementary.pdf]

Supporting information

Abbreviations:

A	absorbance
BuOH	*n*-butanol
c	concentration
CA	chlorogenic acid
CAE	chlorogenic acid equivalents
DCM	dichloromethane
DPPH	2,2-diphenyl-1-picrylhydrazyl
dw	dry weight
EtOAc	ethyl acetate
GA	gallic acid
GAE	gallic acid equivalents
MW	molecular weight
TMS	trimethylsilyl
t_R	retention time
SD	standard deviation

Part I: Folin-Ciocalteu assay

Table S1.1. Calibration data for the Folin-Ciocalteu assay obtained with gallic acid.

c [µg/mL]	A_{765nm}	A_{Blank}	Mean	SD
55	0.416	0.334	0.315	0.019
	0.377	0.295		
	0.398	0.316		
36.7	0.329	0.247	0.227	0.028
	0.289	0.207		
	0.406	0.323*		
27.5	0.242	0.16	0.151	0.009
	0.224	0.142		
	0.232	0.15		
13.8	0.189	0.107	0.084	0.023
	0.166	0.084		
	0.143	0.061		
6.9	0.123	0.041	0.031	0.009
	0.109	0.027		
	0.106	0.024		
3.4	0.091	0.009	0.006	0.004
	0.084	0.002		
	0.089	0.007		

*Value was not considered in calculation.

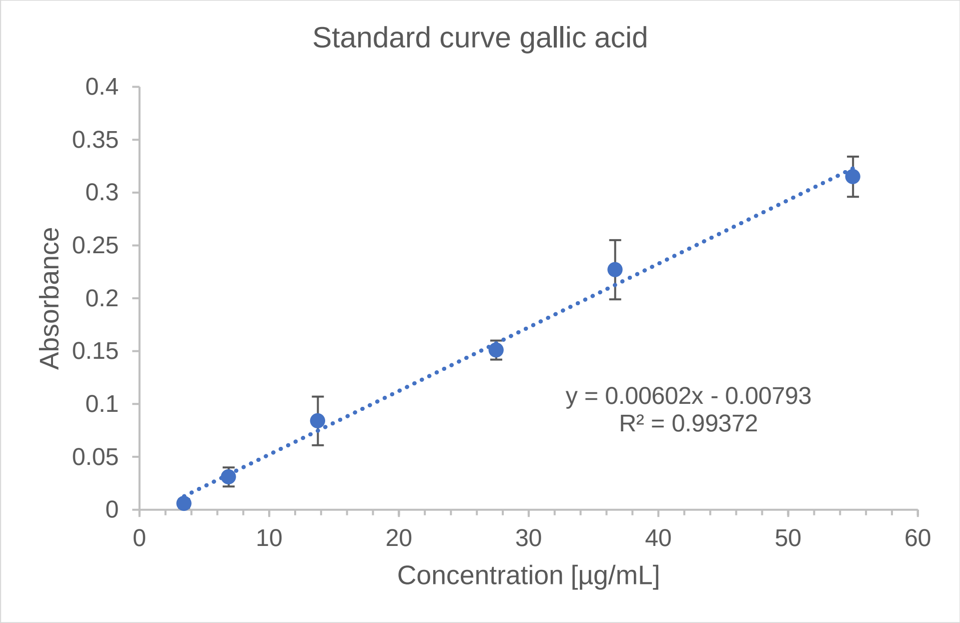

Table S1.2. Calibration data for the Folin-Ciocalteu assay obtained with chlorogenic acid.

c [µg/mL]	A_{765nm}	A_{Blank}	Mean	SD
160	0.639	0.509	0.493	0.035
	0.583	0.453		
	0.647	0.517		
80	0.344	0.214	0.238	0.04
	0.345	0.215		
	0.414	0.284		
53.3	0.277	0.147	0.153	0.029
	0.257	0.128		
	0.314	0.185		
40	0.236	0.106	0.100	0.006
	0.225	0.095		
	0.229	0.099		
20	0.232	0.102	0.061	0.037
	0.164	0.034		
	0.175	0.046		
10	0.116	-0.014	0.001	0.025
	0.117	-0.012		
	0.16	0.03		

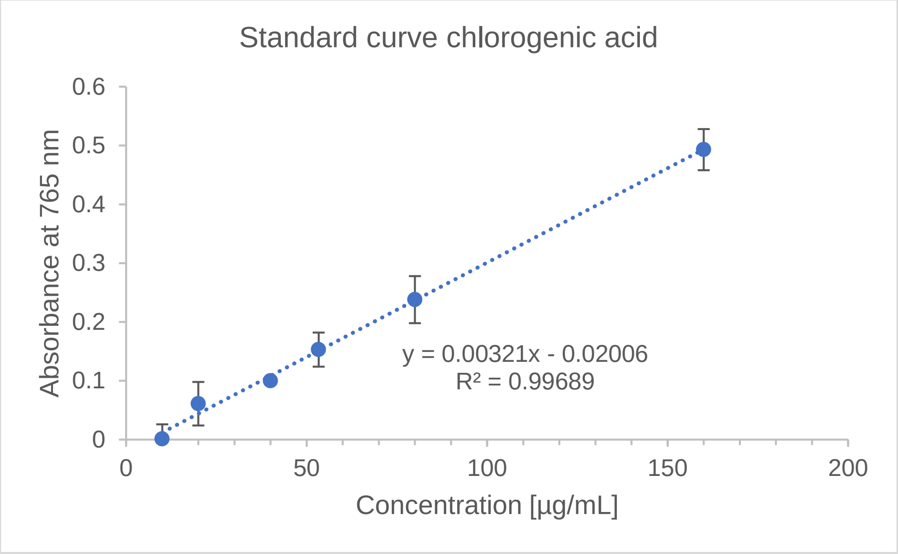

Table S1.3. Total phenolic content of *G. sempervirens* DCM, EtOAc and BuOH extracts (250 µg/mL) calculated as µg gallic or chlorogenic acid equivalents per mg dry weight.

Extract	A_{Blank}	µg GA/ mL	µg GA/ mg dw	Mean	SD	µg CA/ mL	µg CA/ mg dw	Mean	SD
DCM	0.31	52.8	211.2	207.1	5.8	102.8	411.3	411.1	15.6
	0.303	51.6	206.6			100.6	402.6		
	0.288	49.2	196.6			96.0	383.9		
EtOAc	0.394	66.8	267.1	274.0	18.8	129.0	516.0	536.6	41.5
	0.366	62.1	248.5			120.3	481.1		
	0.385	65.3	261.1			126.2	504.7		
BuOH	0.2	34.5	138.2	142.9	10.6	68.6	274.2	290.7	22.7
	0.196	33.9	135.5			67.3	269.2		
	0.215	37.0	148.1			73.2	292.9		

Part II: DPPH assay

Table S2.1. Calibration data for the DPPH radical scavenging assay obtained with trolox.

c [µg/mL]	A_{516nm}	A_{Blank}	Mean	SD	DPPH scavenged [%]
110	0.244<br>0.232<br>0.238	0.164<br>0.152<br>0.158	0.158	0.006	83.1
55	0.636<br>0.649<br>0.639	0.556<br>0.570<br>0.559	0.562	0.007	39.8
27.5	0.828<br>0.832<br>0.846	0.748<br>0.752<br>0.767	0.756	0.01	19.1
13.8	0.928<br>0.930<br>0.935	0.848<br>0.850<br>0.856	0.851	0.004	8.9
6.9	0.982<br>0.983<br>0.987	0.902<br>0.903<br>0.907	0.904	0.003	3.2
3.4	0.995<br>0.999<br>1.004	0.915<br>0.920<br>0.924	0.92	0.004	1.5
1.7	1.007<br>1.013<br>1.021	0.928<br>0.934<br>0.941	0.934	0.007	0.0

Table S2.2. DPPH radical scavenging capacity of the DCM extract at different concentrations.

c [µg/mL]	A_{516nm}	A_{Blank}	Mean	SD	DPPH scavenged [%]
500	0.673<br>0.677<br>0.674	0.593<br>0.597<br>0.594	0.595	0.002	36.3
250	0.821<br>0.818<br>0.804	0.741<br>0.738<br>0.725	0.735	0.009	21.3
125	0.897<br>0.902<br>0.905	0.817<br>0.822<br>0.825	0.822	0.004	12.0
62.5	0.954<br>0.953<br>0.961	0.874<br>0.874<br>0.881	0.876	0.004	6.2
31.3	0.991<br>0.986<br>0.983	0.911<br>0.907<br>0.903	0.907	0.004	2.9

c [µg/mL]	A_{516nm}	A_{Blank}	Mean	SD	DPPH scavenged [%]
15.6	1.004<br>1.003<br>1.009	0.924<br>0.924<br>0.929	0.926	0.003	0.9
7.8	1.015<br>1.016<br>1.009	0.935<br>0.936<br>0.929	0.934	0.004	0.0

Table S2.3. DPPH radical scavenging capacity of the ethyl acetate extract at different concentrations.

c [µg/mL]	A_{516nm}	A_{Blank}	Mean	SD	DPPH scavenged [%]
500	0.132<br>0.132<br>0.566	0.052<br>0.053<br>0.487	0.053	0.001	94.4
250	0.425<br>0.424<br>0.428	0.346<br>0.344<br>0.348	0.346	0.002	63.0
125	0.658<br>0.661<br>0.663	0.578<br>0.581<br>0.584	0.581	0.003	37.8
62.5	0.823<br>0.818<br>0.826	0.743<br>0.738<br>0.747	0.743	0.004	20.4
31.3	0.909<br>0.912<br>0.916	0.829<br>0.832<br>0.836	0.833	0.003	10.8
15.6	0.967<br>0.966<br>0.971	0.887<br>0.886<br>0.891	0.888	0.003	4.9
7.81	0.996<br>0.992<br>0.999	0.916<br>0.912<br>0.919	0.916	0.004	1.9

Table S2.4. DPPH radical scavenging capacity of the *n*-butanol extract at different concentrations.

c [µg/mL]	A_{516nm}	A_{Blank}	Mean	SD	DPPH scavenged [%]
500	0.497	0.418	0.413	0.007	55.8
	0.495	0.416			
	0.485	0.405			
250	0.76	0.681	0.671	0.01	28.2
	0.753	0.673			
	0.74	0.661			
125	0.871	0.792	0.785	0.007	16.0
	0.865	0.786			
	0.857	0.777			
62.5	0.939	0.859	0.857	0.002	8.2
	0.936	0.856			
	0.935	0.855			
31.3	0.975	0.895	0.892	0.004	4.5
	0.974	0.894			
	0.967	0.888			
15.6	1.009	0.929	0.920	0.008	1.5
	0.998	0.918			
	0.993	0.914			
7.8	1.011	0.931	0.926	0.005	0.9
	1.001	0.921			
	1.006	0.927			

Part III: GC-MS analyses

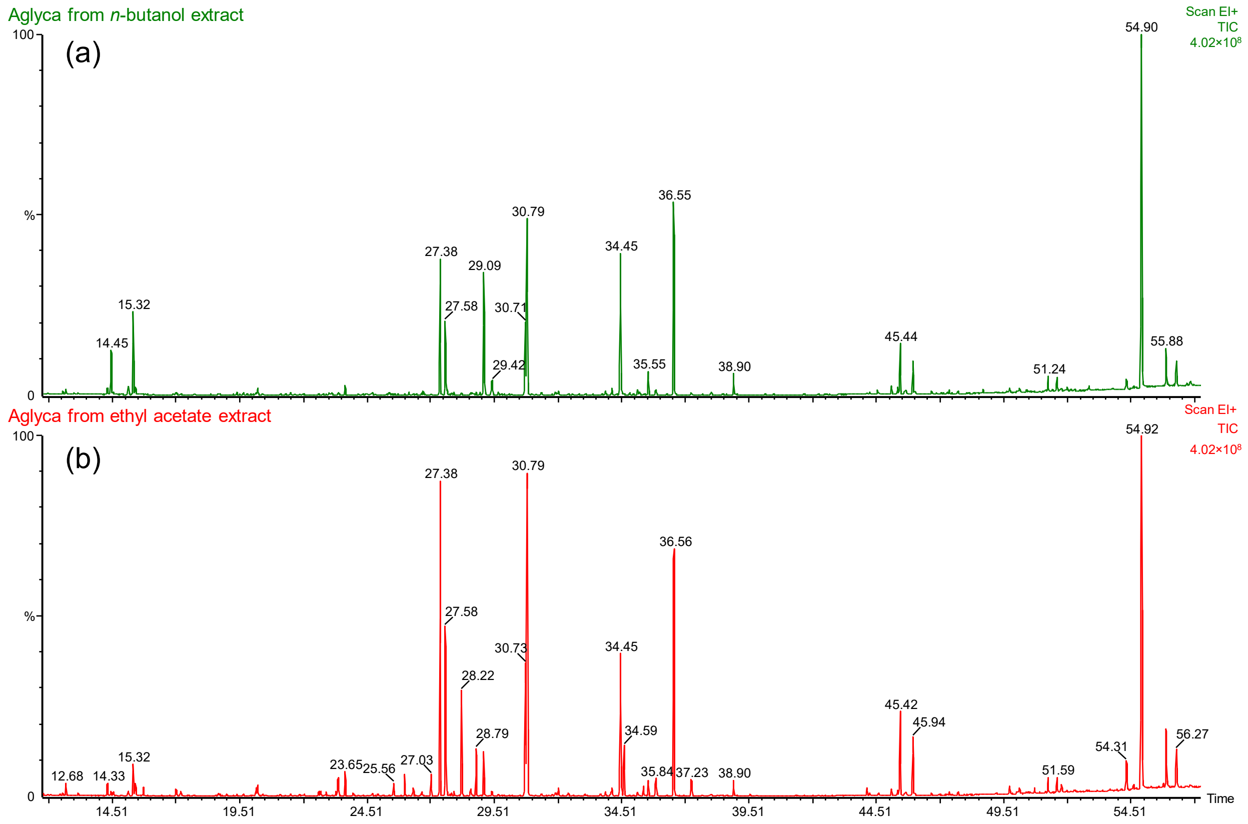

Figure S3.1. GC-MS total ion current chromatograms of hydrolyzed (a) *n*-butanol and (b) ethyl acetate extracts after silylation.

Table S3.1. Aglycones detected in ethyl acetate and *n*-butanol extracts after hydrolysis and silylation using GC-MS. Compound assignment was achieved by comparison with the NIST database (match factor >800).

t_R [min]	Constituent (TMS derivative)	MW [Da]	Fragment *m/z* (Intensity %)
12.7	Benzoic acid	194.3	194 (3), 179 (100), 135 (57), 105 (82), 77 (60)
14.3	Succinic acid	262.4	247 (11), 147 (100), 73 (35)
14.5	3-Methylfurancarboxylic acid	198.3	183 (100), 169 (16), 139 (12), 109 (47), 73 (47)
15.3	Linalool	226.4	226 (9), 143 (100), 94 (13), 81 (31), 73 (74)
23.4	*m*-Salicylic acid	282.5	282 (14), 267 (22), 193 (16), 73 (100)
23.7	*p*-Salicylic acid	282.5	282 (22), 267 (100), 223 (81), 193 (62), 73 (80)
25.6	Suberic acid	318.6	303 (58), 287 (17), 213 (30), 147 (71), 117 (22), 73 (100), 69 (44), 55 (66)
26.0	Suberic acid	318.6	303 (32), 213 (20), 147 (50), 73 (100)
27.0	2,5-Dimethoxy-phenylacetic acid	268.4	268 (1), 253 (17), 209 (20), 134 (18), 105 (33), 73 (100)
27.4	Vanillic acid	312.1	312 (57), 297 (100), 282 (34), 267 (67), 253 (51), 223 (51), 126 (31), 73 (49)
27.6	Gentisic acid	370.6	370 (4), 355 (100), 73 (60)
28.2	Azelaic acid	332.6	317 (51), 201 (52), 147 (26), 129 (31), 117 (29), 73 (100), 55 (48)
28.8	Protocatechuic acid	370.6	370 (49), 355 (26), 311 (21), 193 (100), 73 (53)
29.1	Citral	152.2	255 (18), 152 (32), 107 (22), 84 (51), 75 (52), 73 (41), 69 (100)

t_R [min]	Constituent (TMS derivative)	MW [Da]	Fragment *m/z* (Intensity %)
29.4	Farnesol	294.5	255 (43), 152 (27), 107 (25), 93 (23), 84 (47), 75 (68), 73 (55), 69 (100)
30.7	Pyrogallol	342.7	329 (27), 239 (29), 209 (18), 147 (46), 143 (34), 119 (28), 103 (28), 73 (100)
30.8	Syringic acid	342.5	342 (62), 327 (100), 312 (76), 297 (66), 253 (44), 149 (32), 141 (29), 73 (86)
34.5	Scopoletin	264.3	264 (48), 234 (100), 206 (37), 191 (8), 176 (10), 73 (36)
34.6	Hydroxy-dihydroferulic acid	428.3	428 (46), 297 (86), 73 (100)
35.6	Ferulic acid	338.5	338 (76), 323 (52), 308 (51), 293 (36), 249 (47), 147 (28), 73 (100)
35.8	Esculetin	322.5	322 (35), 307 (30), 73 (100)
36.6	Caffeic acid	396.7	396 (71), 381 (19), 219 (100), 191 (16), 73 (79)
37.2	Linoleic acid	352.3	352 (32), 337 (100), 73 (84)
38.9	Sinapinic acid	368.6	368 (84), 353 (46), 338 (100), 323 (29), 279 (24), 162 (25), 73 (100)
45.4	Unknown		560 (41), 545 (8), 471 (17), 306 (24), 219 (12), 73 (100)
45.9	Unknown		560 (38), 545 (8), 471 (19), 307 (7), 219 (6), 73 (100)
51.2	Vanillylmandelic acid	414.7	401 (1), 297 (100), 73 (49)
51.6	Vanillylmandelic acid	414.7	401 (1), 297 (100), 73 (54)
54.3	Trihydroxyphenylacetic acid	400.7	562 (8), 532 (7), 472 (44), 442 (11), 73 (100),
54.9	Chlorogenic acid	787.4	419 (2), 397 (6), 345 (61), 307 (33), 255 (45), 219 (16), 191 (11), 147 (26), 73 (100)
55.9	Unknown		489 (5), 324 (16), 307 (85), 255 (51), 219 (14), 147 (16), 73 (100)
56.3	Unknown		473 (3), 447 (8), 345 (47), 307 (64), 255 (24), 147 (21), 73 (100)

Deviations from retention times of Table 1 (main manuscript) are due to application of a new GC column.

Table S3.2. GC-MS data of volatile compounds extracted with diethyl ether from aqueous fermented samples. Compound assignment was achieved by comparison with the NIST database (match factor >800).

t_R [min]	Constituent (not silylated)	MW [Da]	Fragment *m/z* (Intensity %)
5.4	Valeric acid	102.1	102 (6), 73 (44), 60 (100), 55 (16)
6.6	Caproic acid	116.2	87 (12), 73 (56), 60 (100), 55 (19)
7.9	Ethyl-3-methyl-butenoate	128.1	128 (51), 113 (58), 95 (44), 83 (100), 67 (65), 55 (83)
8.1	2-Furancarboxylic acid	112.1	112 (100), 95 (61), 55 (10)
9.3	Menthol isomer	156.3	142 (18), 113 (100), 95 (45), 67 (36), 55 (64)
10.6	Benzoic acid	122.1	122 (84), 106 (11), 105 (100), 77 (91)
11.1	2-Acetylphenol	136.1	136 (43), 121 (100), 93 (19), 65 (20), 56 (11)
11.7	3-Propylcyclohex-1-ene	124.2	124 (20), 81 (100), 71 (30), 53 (17)
13.5	1-Methylethylidene cyclohexane	124.2	124 (63), 109 (12), 93 (32), 81 (100), 67 (23), 55 (38)
14.2	*o*-Salicylic acid	138.1	138 (61), 120 (90), 92 (100), 64 (32), 63 (22)
15.0	3,7-Dimethyloct-1-ene	140.3	100 (44), 84 (17), 69 (29), 55 (100)
16.4	2,4-Octadienoic acid	140.1	140 (22), 97 (76), 81 (100), 79 (66), 69 (53), 55 (54)
16.5	γ-Amylbutyrolactone	156.2	85 (100), 55 (11)
16.9	4-Ethylcatechol	138.2	138 (30), 123 (100), 77 (15)

t_R [min]	Constituent (not silylated)	MW [Da]	Fragment *m/z* (Intensity %)
18.7	2-Hydroxy-6-methylbenzaldehyde	136.1	136 (100), 89 (34), 79 (16), 63 (13)
20.5	*p*-Salicylic acid	138.1	138 (51), 121 (100), 93 (26), 65 (18)
20.7	Butylhydroxytoluol*	220.4	220 (24), 205 (100), 177 (15), 145 (14), 57 (17)
22.5	Isovanillic acid	168.1	168 (100), 153 (100), 125 (33), 97 (34), 79 (11), 52 (12)
23.5	3,4,5-Trimethoxyphenol	184.2	184 (73), 169 (100), 141 (52), 126 (27), 111 (27), 69 (43)
23.9	Azelaic acid	188.2	168 (39), 152 (40), 124 (22), 111 (32), 83 (53), 73 (36), 69 (56), 60 (55), 55 (100)
24.9	Oxo-α-ionol	208.3	152 (11), 134 (12), 109 (20), 108 (100), 91 (19)
26.6	7,8-Dihydro-3-oxo-α-ionol	210.3	210 (23), 177 (27), 150 (54), 135 (100), 123 (84), 108 (80), 93 (92), 79 (52)
26.8	Dehydrogeosinin or 4-ethylcamphor	180.2	180 (96), 151 (18), 122 (19), 109 (100), 81 (50), 69 (34), 53 (55)
27.1	Protocatechuic acid	154.1	154 (82), 137 (100), 109 (29), 81 (23), 79 (6), 53 (17)
29.4	Syringic acid	198.1	198 (100), 183 (43), 127 (28), 109 (21), 81 (16)
30.7	Ferulic acid	194.2	194 (100), 179 (34), 133 (23), 77 (30)
31.6	Dimethyloctahydroiso-benzofuran-4-ol	170.2	170 (15), 140 (18), 107 (32), 95 (32), 81 (100), 69 (30), 55 (44)
33.5	Scopoletin	192.2	192 (100), 177 (59), 164 (28), 149 (58), 121 (29), 69 (37)
34.6	Caffeic acid	180.2	180 (100), 163 (27), 134 (58), 89 (32), 77 (25)
36.2	Unknown		222 (100), 207 (52), 179 (20), 123 (36), 95 (23), 79 (19)
36.3	Unknown		222 (100), 207 (23), 179 (23), 123 (15), 95 (12), 79 (12)

*Stabilizing agent in diethylether.

Part IV: RP-HPLC-DAD-ESI-MS^n analyses

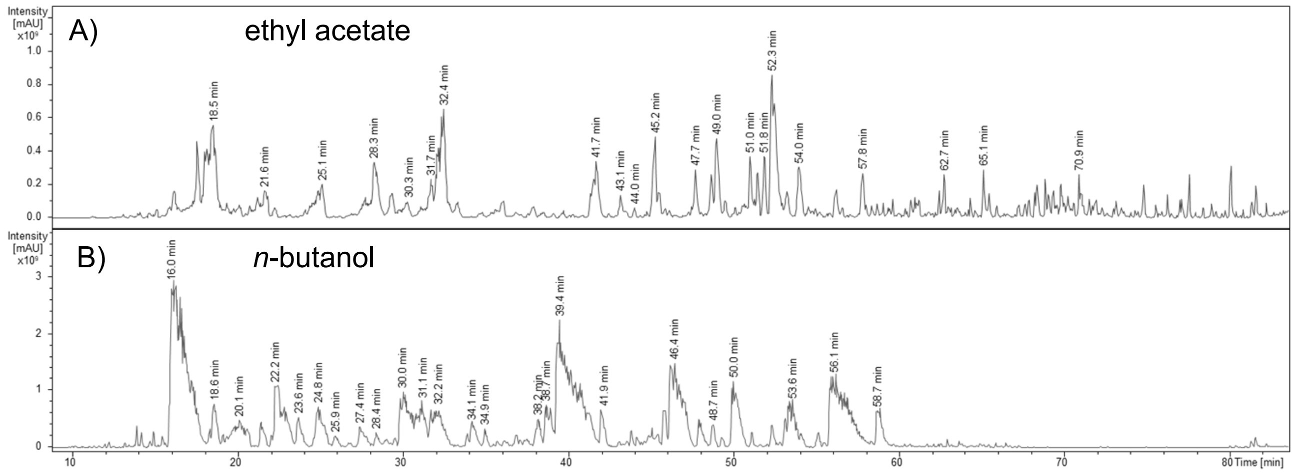

Figure S4.1. HPLC-DAD-ESI-MS^n base peak chromatograms of A) ethyl acetate and B) *n*-butanol extracts of *G. sempervirens* roots and rhizomes recorded in positive ionization mode.

Table S4.1. HPLC-DAD-MS^n characteristics of individual secondary metabolites in ethyl acetate and *n*-butanol extracts from *G. sempervirens* roots and rhizomes obtained in positive ionization mode.

Substance	t_R [min]	extract		UV [nm]	Mass spectrometric data *m/z*			Ref.
		EtOAc	BuOH		MS^1	MS^2	MS^3	
Gelsemine	16.0		X	208, 252, 250, 325	**323**	274, **236,** 196, 190		[1,2]
5-O-Caffeoylquinic acid	18.5	X	X	218, 236, 312, 328	**355**, 163	163	145	[3]
Koumidine	20.1		X	222, 288	387, **295**	297, 265		[2]
Caffeic acid	21.6	X	X	226, 302	**181**	163, 137, 93		
Hydroxygelselegine	22.2		X	224, 290	**375**	**344**	315, 270, 217, 176, 148	[2]
Unknown	23.6		X	230, 322	**391**	**360**, 331	341, 331, 313, 244	
15-Hydroxy-humantenine	24.8		X	-	**371**	**340**	326, 295, **277**, 246	[2]
Dihydroferulic or dimethylphenylacetic acid	25.1	X		218, 316	**197**	**179**		[4]
Koumidine	25.9		X	-	**295**	277, **254,** 225, 171, 144, 132		[2]
19-(Z)-Akuammidine	27.4<br>28.3	 X	X		**353**	333, **323**, 166, 157		[2]
19-(Z)-Anhydrovobasinediole	28.4		X	-	**309**	291	175	[1]
Gelsevirine	30.0		X	216, 324	367, **353**	**322**	294, 289	[2]
Feruloylquinic acid	30.3	X		238, 326	369, **177**	**145**	89	[5]
Gelsempervine A	31.1<br>32.2		X	226, 264, 340	**383,** 193	**180**	**165,** 148	[1,2]
Gelsevirine	31.7	X		204, 228, 255sh, 295sh, 342	**353,** 193	**322**	307, 304, 391, 189, 281, 264, 261, 233, 223, 251, 132	[1,2]

Substance	t_R [min]	extract		UV [nm]	Mass spectrometric data *m/z*			Ref.
		EtOAc	BuOH		MS^1	MS^2	MS^3	
Scopoletin	32.4	X		207, 228, 296, 342	**193**	**173**		[6]
1-Methoxy-21-oxo-gelsemine	34.1		X	-	**367**	**334**, 180	304	
Gelsedine	38.2		X	226, 288	**329**	**296**	256 224 162	[1]
Sarpagan-16-carboxylic acid, 17-oxo-, methyl ester	38.7		X	252, 304	**351**	**333**	319	
N-Methylgelsedilam	39.4		X	208, 258	351, **329**	**298,** 269	278, 268, 242	[1]
	41.7	X						
Gelegamine C	41.9		X	224	**389**	**339**	226, **175**	[2]
	43.1	X		232, 286	285, **245**	227, 225, **171**	125	
Hydroxygelsenicine	44.0	X		222, 260sh, 286	**343**	325, 181		[1]
Unknown	45.2	X		224, 288	**535**	343, **181**	153	
Gelsemicine	46.4		X	234	**359**	328	298, 245, 166, 152	[1,2]
Gelsemicine	47.7	X		234	**359**	**328**	298, 284, 236, 202	[1,2]
14-Acetoxygelsenicine or 11-Methoxy-humantenine	48.7		X	230, 286, 342	**385,** 371, 315	**354**	321	[1,2]
Dicaffeoylquinic acid isomer	49.0	X		222, 235, 300sh, 326	517, **499**	**319**	299, 163	[3]
11-Hydroxyhumantenine or Humantenirine	50.0		X	224, 290, 326	**371**	**340**	175, 105	[1,2]
	51.0	X						
Dicaffeoylquinic acid isomer	51.8	X		232, 292	535, **517**	**337**	181	[3]
Dicaffeoylquinic acid isomer	52.3	X		220, 240, 300sh, 326	**517**, 499	319, **163**	145, 117	[3]
Tabersonine	53.6		X	234, 322	**337**	305, **180**		[7]
Gelsempervine	54.0	X		234, 322	**425**	**383**, 305, 206	208, 173, 146	[1]
Sempervirin	56.1		X	240, 292, 336, 382	**273**	**257**	185, 159	[1,2]
Unknown	57.8	X		234, 324	561, **543**	363, **207**	175	
Ourouparine	58.7		X	204, 236, 284, 340	**329,** 273	314, 295, 283		[8]
Gelse-norursane A	62.7	X		232, 326	**503**	**439**	421	[1]
Trihydroxy-octadecenoic acid isomer	65.1	X		-	**294**	**277**	131, 107	[4]
Gelse-norursaneE	70.9	X		-	**471**	**453**	407, 217	[1]

Ions displayed in **bold** were isolated for further fragmentation.

References

1. Liu, Y.-C.; Lin, L.; Cheng, P.; Sun, Z.-L.; Wu, Y.; Liu, Z.-Y. Fingerprint analysis of *Gelsemium elegans* by HPLC followed by the targeted identification of chemical constituents using HPLC coupled with quadrupole-time-of-flight mass spectrometry. *Fitoterapia* **2017**, *121*, 94–105, doi:10.1016/j.fitote.2017.07.002.
2. Wang, J.; Zhang, J.; Zhang, C.; Sun, X.; Liao, X.; Zheng, W.; Yin, Q.; Yang, J.; Mao, D.; Wang, B.; et al. The qualitative and quantitative analyses of *Gelsemium elegans*. *J. Pharm. Biomed. Anal.* **2019**, *172*, 329–338, doi:10.1016/j.jpba.2019.05.015.
3. Clifford, M.N.; Johnston, K.L.; Knight, S.; Kuhnert, N. Hierarchical scheme for LC-MS^n identification of chlorogenic acids. *J. Agric. Food Chem.* **2003**, *51*, 2900–2911, doi:10.1021/jf026187q.

4. Jiménez-Sánchez, C.; Lozano-Sánchez, J.; Rodríguez-Pérez, C.; Segura-Carretero, A.; Fernández-Gutiérrez, A. Comprehensive, untargeted, and qualitative RP-HPLC-ESI-QTOF/MS[2] metabolite profiling of green asparagus (*Asparagus officinalis*). *J. Food Comp. Anal.* **2016**, *46*, 78–87, doi:10.1016/j.jfca.2015.11.004.
5. Abu-Reidah, I.M.; Arráez-Román, D.; Segura-Carretero, A.; Fernández-Gutiérrez, A. Extensive characterisation of bioactive phenolic constituents from globe artichoke (*Cynara scolymus* L.) by HPLC-DAD-ESI-QTOF-MS. *Food Chem.* **2013**, *141*, 2269–2277, doi:10.1016/j.foodchem.2013.04.066.
6. Liu, Y.-C.; Xiao, S.; Yang, K.; Ling, L.; Sun, Z.-L.; Liu, Z.-Y. Comprehensive identification and structural characterization of target components from *Gelsemium elegans* by high-performance liquid chromatography coupled with quadrupole time-of-flight mass spectrometry based on accurate mass databases combined with MS/MS spectra. *J. Mass Spectrom.* **2017**, *52*, 378–396, doi:10.1002/jms.3937.
7. Que, W.; Chen, M.; Yang, L.; Zhang, B.; Zhao, Z.; Liu, M.; Cheng, Y.; Qiu, H. A network pharmacology-based investigation on the bioactive ingredients and molecular mechanisms of *Gelsemium elegans* Benth against colorectal cancer. *BMC Complement. Med. Ther.* **2021**, *21*, 99, doi:10.1186/s12906-021-03273-7.
8. Kogure, N.; Someya, A.; Urano, A.; Kitajima, M.; Takayama, H. Total synthesis and full NMR assignment of ourouparine, a yohimbane-type indole alkaloid isolated from *Gelsemium sempervirens*. *J. Nat. Med.* **2007**, *61*, 208–212, doi:10.1007/s11418-006-0125-4.

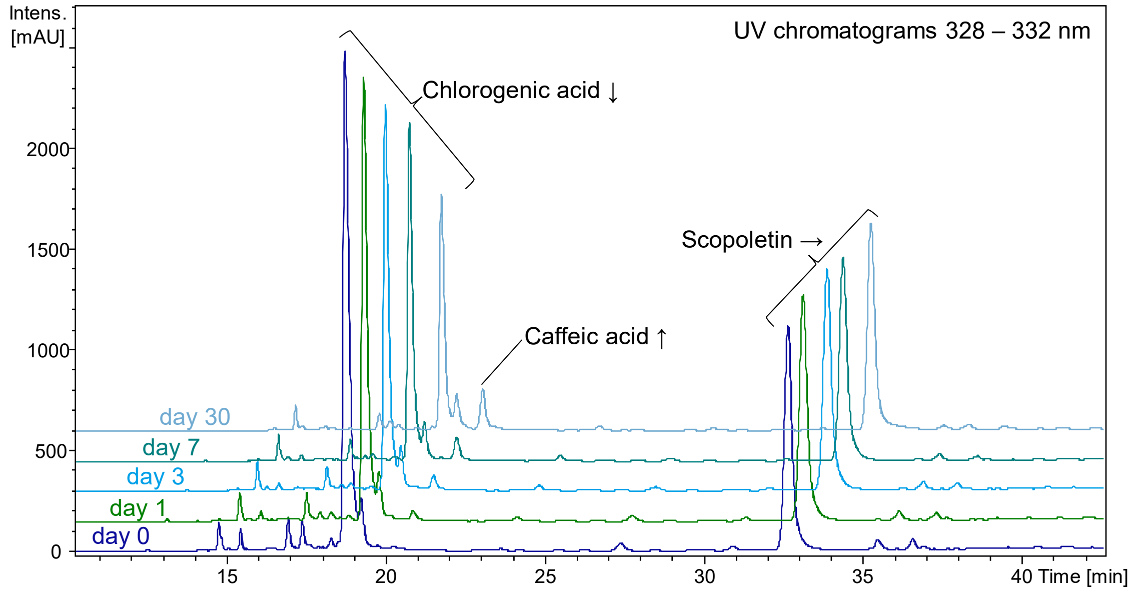

Figure S4.2. Stacked display of UV chromatograms (328 – 332 nm) of aqueous fermented *G. sempervirens* extracts within 30 days of fermentation.
